# Transcriptional Regulation of lncRNA Genes by Histone Modification in Alzheimer's Disease

**DOI:** 10.1155/2016/3164238

**Published:** 2016-10-16

**Authors:** Guoqiang Wan, Wenyang Zhou, Yang Hu, Rui Ma, Shuilin Jin, Guiyou Liu, Qinghua Jiang

**Affiliations:** ^1^School of Computer Science and Technology, Harbin Institute of Technology, Harbin 150001, China; ^2^Department of Mathematics, Harbin Institute of Technology, Harbin 150001, China

## Abstract

Increasing studies have revealed that long noncoding RNAs (lncRNAs) are not transcriptional noise but play important roles in the regulation of a wide range of biological processes, and the dysregulation of lncRNA genes is associated with disease development. Alzheimer's disease (AD) is a chronic neurodegenerative disease that usually starts slowly and gets worse over time. However, little is known about the roles of lncRNA genes in AD and how the lncRNA genes are transcriptionally regulated. Herein, we analyzed RNA-seq data and ChIP-seq histone modification data from CK-p25 AD model and control mice and identified 72 differentially expressed lncRNA genes, 4,917 differential peaks of H3K4me3, and 1,624 differential peaks of H3K27me3 between AD and control samples, respectively. Furthermore, we found 92 differential peaks of histone modification H3K4me3 are located in the promoter of 39 differentially expressed lncRNA genes and 8 differential peaks of histone modification H3K27me3 are located upstream of 7 differentially expressed lncRNA genes, which suggest that the majority of lncRNA genes may be transcriptionally regulated by histone modification in AD.

## 1. Introduction

Alzheimer's disease (AD) is a neurodegenerative disease with unknown etiology [1–3]. The main clinical manifestation is intelligence damage. In addition, it is the cause of 60% to 70% of cases of dementia. AD often begins in people over 65 years of age, and it affects approximately 6% of people aged 65 years and older [4]. There are about 48 million persons suffering from AD around the world in 2015, and dementia resulted in about 486,000 deaths in 2010 [5].

Long noncoding RNAs (lncRNAs) are non-protein-coding transcripts longer than 200 nucleotides in length. Thousands of human and mouse lncRNAs have been identified and emerging studies have revealed that lncRNAs play important roles in a wide range of biological processes and diseases [6–12]. Many studies have demonstrated that lncRNAs play crucial roles in the regulation of gene expression at epigenetic, transcriptional, and posttranscriptional level [13]. However, little is known about how lncRNA genes are transcriptionally regulated [14] in disease such as AD.

In this paper, we analyzed RNA-seq data and ChIP-seq histone modification data from control mice and CK-p25 AD model at 2 weeks after induction of neurodegeneration and checked whether lncRNA genes are transcriptionally regulated by histone modification in AD.

## 2. Materials and Methods

### 2.1. RNA-seq and ChIP-seq Data in AD and Control

The RNA-seq and ChIP-seq data were downloaded from GEO database with ID GSE65159 [15]. There are three control samples and three AD mice model samples at 2 weeks after induction of neurodegeneration. The histone modification marks include H3K4me3 and H3K27me3.

### 2.2. Identifying Differentially Expressed lncRNA Genes between AD and Control

We used RNA-seq data to evaluate gene expression on control mice and CK-p25 Alzheimer's disease model. We used the mm10 reference sequence to build an index by Bowtie2-build [16]; the mm10 reference sequence was downloaded from UCSC. Next the RNA-seq data are mapped to the mm10 reference sequence with TopHat2 [17] by default parameters. Cufflinks [18] was used to assemble the outcome of mapping and evaluate gene expression index. The lncRNA annotation was downloaded from GENCODE database, and differentially expressed lncRNA genes were identified by Cuffdiff with default parameters, a component of Cufflinks software.

### 2.3. Identifying Differential Histone Modification Peaks

To explore whether differentially expressed lncRNAs between AD and control are regulated by histone modification or not, we identified differential histone modification regions by analyzing the ChIP-seq data of histone marks H3K4me3 and H3K27me3 in AD and control. We firstly mapped the ChIP-seq data to the mm10 reference sequence by Bowtie2 software with default parameters. Then we used MACS2-callpeak [19] to identify the peaks of histone modification regions in the control mice and CKp25 Alzheimer's disease model [20], respectively. Finally, MACS2-bdgdiff is used to identify significantly differential histone modification regions between the control and AD.

### 2.4. Linking the Differential lncRNA Genes with the Differential Histone Modification Peaks Based on the Genomic Position

After identifying differential histone modification regions and differentially expressed lncRNA genes, we investigated whether the differential histone modification regions are located in the regulatory regions of the differential lncRNA genes. Herein, the regulatory regions are defined as 10 kbp upstream to 1 kbp downstream of transcriptional start site (TSS) of each differentially expressed lncRNA gene.

## 3. Results

### 3.1. Differentially Expressed lncRNA Genes between AD and Control Samples

By analyzing three AD and control RNA-seq data, we identified 72 significantly differentially expressed lncRNA genes with the BH-adjusted *p* value < 0.05 and fold change >2 (Supplementary Table  1, in Supplementary Material available online at http://dx.doi.org/10.1155/2016/3164238).

### 3.2. Differential Histone Modification Peaks between AD and Control Samples

We analyzed ChIP-seq histone modification data from CK-p25 AD model and control mice and identified 4,917 differential peaks of H3K4me3 and 1,624 differential peaks of H3K27me3 between AD and control samples, respectively.

### 3.3. Differential Histone Modification Peaks Are Located Upstream of Differentially Expressed lncRNA Genes

**Figure 1:**
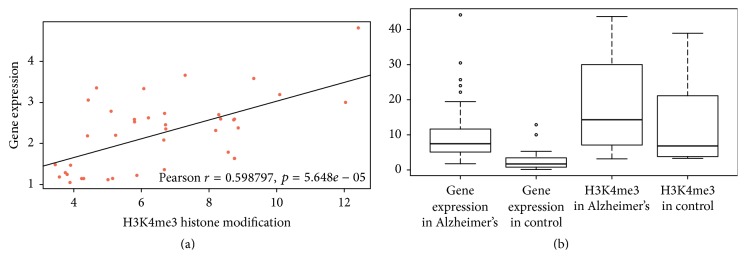
Positive association between expression level of differential lncRNA genes and H3K4me3 modification level in promoters of the differential lncRNA genes. (a) Scatter diagram and a fitting line show the positive association between fold change of lncRNA gene expression and fold change of H3K4me3 modification level in the promoter. (b) Boxplot of expression level of differential lncRNA genes and H3K4me3 modification level in the promoters of the differential lncRNA genes in AD and control samples, respectively, which shows that lncRNA genes with high H3K4me3 level in the promoters have high expression level. The circle in (b) refers to a singular point in statistics, differential from other points. But the singular point has statistical significance, showing the accuracy and objectivity of this article.

**Figure 2:**
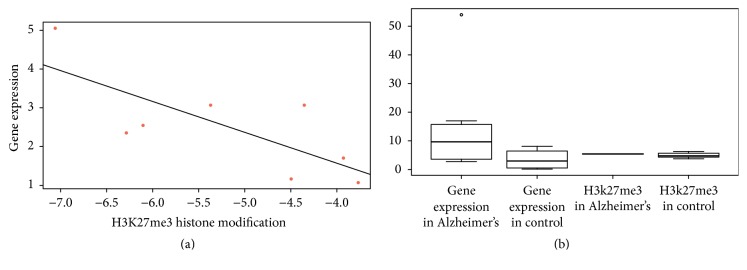
Negative association between expression level of differential lncRNA genes and H3K27me3 modification level in promoters of the differential lncRNA genes. (a) Scatter diagram and a fitting line show the negative association between fold change of expression level of differential lncRNA genes and fold change of H3K27me3 modification level in the promoters. (b) Boxplot of expression level of differential lncRNA genes and H3K27me3 modification level in the promoters of the differential lncRNA genes in AD and control samples, respectively, which shows that lncRNA genes with high H3K27me3 level in the promoters have low expression level. The circle in (b) refers to a singular point in statistics, differential from other points. But the singular point has statistical significance, showing the accuracy and objectivity of this article.

**Figure 3:**
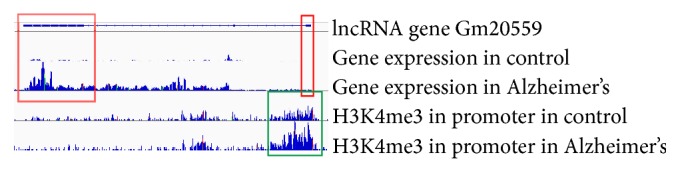
A lncRNA gene named Gm20559 with differential H3K4me3 modification level in the promoter between AD and control samples shows differential expression in exon 1 and exon 3. The red rectangle shows exon 1 and exon 3 regions of differentially expressed lncRNA gene Gm20559. And the green rectangle shows differential H3K4me3 histone modification in the promoter region, which suggests transcriptional regulation of Gm20559 by H3K4me3.

We found that there are 92 H3K4me3 differential histone modification peaks located in the promoters (2 kbp upstream to −1 kbp downstream) of 39 differentially expressed lncRNA genes (Supplementary Table  2) and 8 differential H3K27me3 histone modification peaks located in the region from 10 kb upstream to −1 kb downstream of 7 differentially expressed lncRNA genes. A positive association between histone modification level of H3K4me3 and lncRNA gene expression level is shown in Figure 1, and a negative association between histone modification level of H3K27me3 and lncRNA gene expression level is shown in Figure 2. A case study for the lncRNA gene named Gm20559 was shown in Figure 3, where the lncRNA Gm20559 had differential histone modification of H3K4me3 between AD and control in its promoter region, and exon 1 and exon 3 of Gm20559 are differentially expressed between AD and control. These results suggest that the majority of lncRNA genes (39 + 7)/72 may be transcriptionally regulated by histone modification in AD.

## 4. Discussion

lncRNA is a type of important regulatory RNAs that play critical roles in a wide range of biological processes. However, how the lncRNA genes themselves are transcriptionally regulated remains to be elucidated. In this paper, we used RNA-seq and ChIP-seq data from AD model and control to demonstrate that the majority of lncRNA genes are transcriptionally regulated by histone modification in AD.

As known, a protein-coding gene or lncRNA gene is regulated by many types of factors rather than one factor. Therefore, it sounds reasonable to integrate kinds of factors such as transcription factor, microRNA [21–24], DNA methylation, and histone modification to investigate the transcriptional regulation of lncRNAs in a specific condition such as AD, which will improve our understanding of lncRNA genes in AD.

## Supplementary Material

Differentially expressed lncRNA genes and differential Histone modification of H3K4me3 located in promoter of 39 lncRNA genes.
